# Context-dependent cGAS-STING signaling in solid tumors: biological determinants, clinical implications, and biomarker-guided therapeutic strategies

**DOI:** 10.3389/fimmu.2026.1954279

**Published:** 2026-10-19

**Authors:** Zhenru Wang, Yuerufei Yu, Ying Zhu

**Affiliations:** The First Affiliated Hospital of Zhejiang Chinese Medical University (Zhejiang Provincial Hospital of Chinese Medicine), Hangzhou, Zhejiang, China

**Keywords:** candidate biomarkers, cGAS-STING, chromosomal instability, colorectal cancer, immune checkpoint blockade, STING agonist, tumor microenvironment

## Abstract

Stimulator of interferon genes (STING) agonists are intended to convert cytosolic DNA sensing into productive antitumor immunity, yet their biological effects vary substantially among tumors. Outcomes depend on the cellular compartment engaged, the amplitude and duration of signaling, tumor genotype, the immune architecture of the tumor microenvironment, and drug distribution. A critical distinction must therefore be made between chronic endogenous, tumor-cell-intrinsic cyclic GMP-AMP synthase-stimulator of interferon genes (cGAS-STING) signaling, as may occur in chromosomally unstable tumors, and controlled pharmacological STING agonism. The former can sustain maladaptive inflammatory programs in selected experimental settings, whereas the latter can be delivered transiently and may preferentially activate dendritic cells and other host immune compartments. This narrative review examines canonical STING-TBK1-IRF3/type I interferon signaling and alternative chronic outputs; relates these mechanisms to chromosomal instability, mismatch-repair status, immune surveillance, and immune checkpoint blockade; and evaluates early clinical experience with intratumoral STING agonists. Available trials demonstrate target engagement and generally manageable toxicity, but efficacy has been limited or heterogeneous. Mismatch-repair status, tumor mutational burden, chromosomal instability, baseline interferon activity, STING pathway competence, immune-cell topology, and delivery feasibility should therefore be considered components of a candidate composite biomarker strategy rather than validated predictors. By contrast, STING inhibition in chromosomal-instability-high tumors remains a promising preclinical hypothesis, not an established treatment. Prospective, compartment-resolved biomarker studies are needed to match agonists, delivery systems, combination regimens, or inhibitory approaches to the appropriate tumor state.

## 1 Introduction

The cGAS-STING pathway links cytosolic DNA sensing to innate and adaptive immunity and has therefore become an attractive target in cancer immunotherapy. Wang and colleagues summarized the major classes of STING agonists, delivery platforms, clinical studies, and translational challenges (1). That pharmacological overview also highlights a deeper problem: engaging the same molecular node does not necessarily produce the same biological outcome in every tumor. The consequences of STING activation depend on which cells receive the signal, whether activation is transient or persistent, which downstream programs remain competent, and whether the surrounding tumor microenvironment can convert innate inflammation into effective T-cell immunity. This narrative review examines these context-dependent determinants. We first distinguish chronic endogenous, tumor-cell-intrinsic cGAS-STING signaling from pharmacological STING agonism. We then consider pathway outputs, chromosomal instability, immune surveillance, and tumor microenvironment states across solid tumors, with particular attention to colorectal cancer and responses to immune checkpoint blockade (ICB). Finally, we summarize early clinical evidence and propose a candidate biomarker framework for identifying settings in which STING activation, combination therapy, or, in selected preclinical contexts, pathway inhibition may be biologically rational.

## 2 Two biologically distinct settings of STING activation

### 2.1 Canonical acute signaling and immune priming

Cytosolic double-stranded DNA activates cGAS, which generates 2’3’-cyclic GMP-AMP (cGAMP) and engages endoplasmic reticulum-resident STING. STING trafficking to the Golgi enables recruitment of TANK-binding kinase 1 (TBK1), phosphorylation of interferon regulatory factor 3 (IRF3), and induction of type I interferons and interferon-stimulated genes (2). In dendritic cells, this program supports antigen presentation, cross-priming, and the recruitment of tumor-reactive T cells. Preclinical studies established that host STING-dependent DNA sensing is required for spontaneous recognition of immunogenic tumors and that direct intratumoral STING activation can generate both local and systemic antitumor immunity (3–5).

This canonical STING-TBK1-IRF3/type I interferon axis provides the principal therapeutic rationale for STING agonists. However, antitumor activity cannot be inferred from STING expression alone. It requires a competent signaling cascade, an appropriate antigen-presenting-cell compartment, available tumor antigens, and the capacity to translate innate activation into adaptive immunity. In preclinical models, the magnitude of therapeutic STING activation also shaped the quality of CD8+ T-cell-mediated immunity, indicating that stronger signaling is not necessarily more effective (6).

### 2.2 Chronic endogenous tumor-cell-intrinsic signaling

Endogenous pathway activation is biologically distinct from administration of a defined agonist dose. Genomic instability, micronuclear rupture, mitochondrial DNA leakage, replication stress, and therapy-induced DNA damage can repeatedly expose DNA to cGAS. Micronuclei generated after chromosome-segregation errors or DNA damage are recognized by cGAS and can sustain inflammatory signaling across successive cell cycles (7, 8). In chromosomally unstable tumors, this stimulus may be continuous, tumor-cell intrinsic, and shaped by selection for cells that tolerate or rewire the pathway (9, 10).

Pharmacological agonism, by contrast, can be transient, locally injected, systemically delivered, or targeted through a carrier, and it may engage dendritic cells, macrophages, endothelial cells, or tumor cells in different proportions. Evidence that chronic tumor-cell-intrinsic STING signaling promotes progression in a particular experimental model therefore does not establish that pharmacological STING agonists are pro-tumorigenic. The two settings should be compared mechanistically rather than treated as interchangeable exposures.

### 2.3 Divergent downstream outputs

STING signaling does not produce a single uniform output (11). Acute activation can favor TBK1-IRF3-driven type I interferon production and adaptive immune priming, whereas prolonged or repeatedly selected tumor-cell signaling can include NF-κB-dependent inflammation, stress-response programs, autophagy, cell death, or partial uncoupling from productive interferon signaling. In low-antigenicity tumor models, STING-dependent induction of indoleamine 2,3-dioxygenase promoted immune tolerance rather than tumor rejection (12). Chronic tumor interferon signaling has also been linked to a multigenic resistance program during checkpoint blockade, although this finding does not establish a STING-dependent mechanism in every tumor (13). Treatment resistance and immune exclusion should therefore not be described as generic consequences of persistent DNA sensing; they are context-specific possibilities that require direct validation of both pathway activity and cellular compartment.

### 2.4 Compartment, dose, route, and schedule are part of the mechanism

Intratumoral injection can create a high local concentration and facilitate sampling of an injected lesion, but it is unsuitable for many metastatic distributions and does not ensure immune activation at uninjected sites. Systemic small molecules, nanocarriers, exosome-based platforms, antibody-drug conjugates, and engineered microorganisms differ in biodistribution, cellular uptake, and toxicity. A short pulse in dendritic cells may favor immune priming, whereas prolonged, diffuse, or repeatedly high exposure may induce counter-regulatory cytokines, tissue injury, or adaptation. Route, dose, and schedule should therefore be treated as biological variables rather than operational details (14).

In mouse tumor models, systemically administered, tumor-directed STING agonist antibody-drug conjugates activated malignant and myeloid cells while reducing serum cytokine elevations relative to a free agonist (15). However, controlled extension of dendritic-cell signaling is not equivalent to chronic tumor-intrinsic activation: a dual-STING-activating micelle prolonged antigen presentation and T-cell responses in mice (16). Together with dose-dependent CD8+ T-cell effects (6), these findings favor optimizing exposure kinetics rather than maximizing dose or universally minimizing duration. Comparative studies should therefore measure tissue exposure and cellular uptake alongside systemic cytokines and lymphocyte viability. The two signaling contexts and their main modifiers are shown in **Figure 1**.

**Figure 1:**
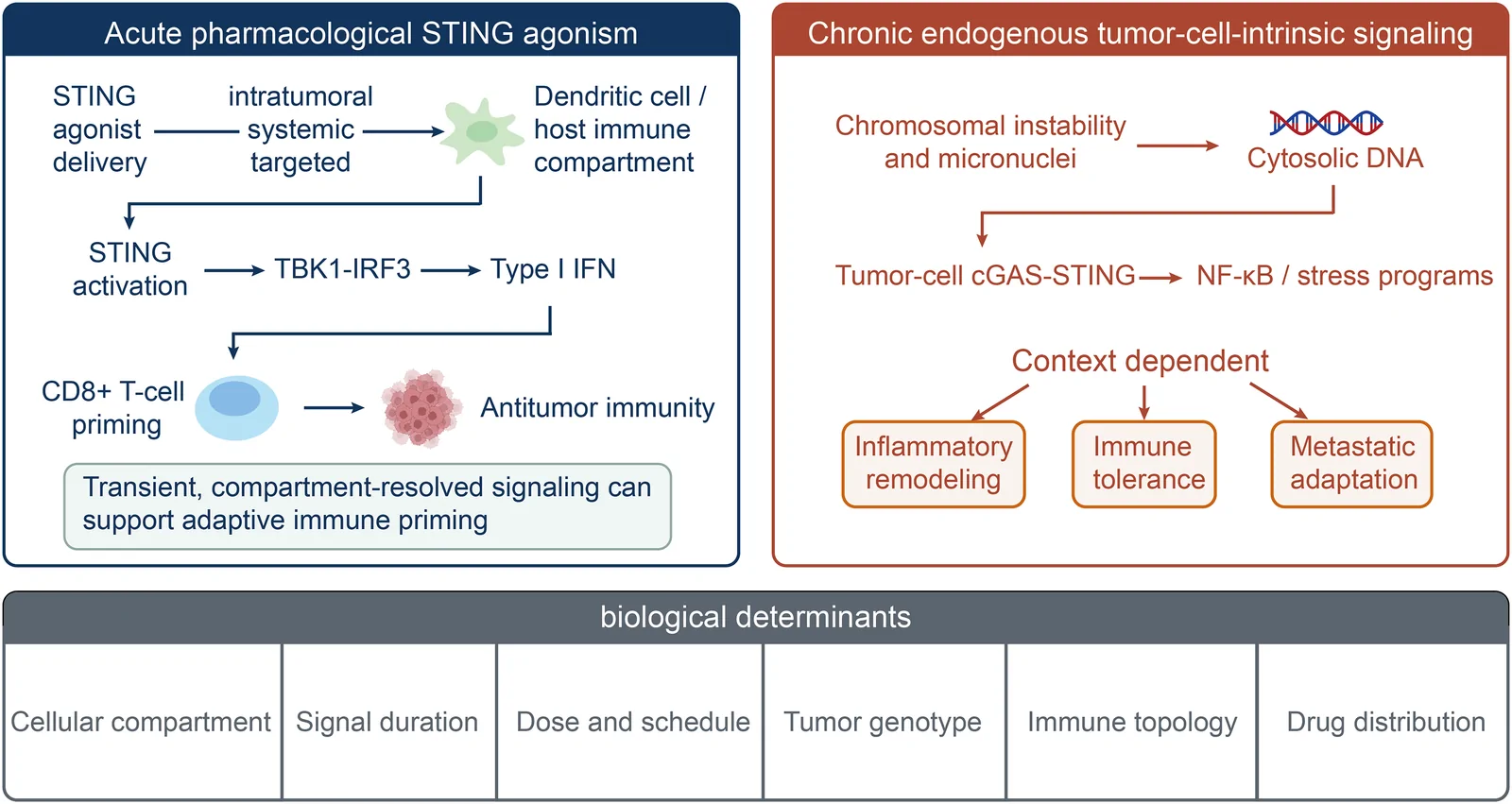
Context-dependent consequences of acute pharmacological STING agonism and chronic endogenous tumor-cell-intrinsic cGAS-STING signaling. cGAS, cyclic GMP-AMP synthase; IFN, interferon; IRF3, interferon regulatory factor 3; NF-kappa B, nuclear factor kappa B; STING, stimulator of interferon genes; TBK1, TANK-binding kinase 1.

## 3 Cellular and tumor microenvironment heterogeneity

### 3.1 Immune and stromal compartments

Dendritic-cell activation is central to the intended immune-priming effect of many STING agonists, but other compartments can redirect the response. In mice, the PolySTING nanoparticle required STING signaling in type 1 conventional dendritic cells (cDC1s) and CCR7-dependent migration to tumor-draining lymph nodes for tumor control (17). Depending on their state, macrophages may produce inflammatory cytokines, support antigen presentation, or reinforce suppressive programs. Macrophage STING also links innate sensing to effector-cell cooperation. In a mouse model of colorectal cancer liver metastasis, macrophage STING activated NLRP3-dependent interleukin-18 and interleukin-1β production and 4-1BBL/4-1BB costimulation with natural killer (NK) cells; myeloid STING loss impaired NK function and increased metastatic burden (18). In mouse colorectal tumors, the prodrug GB2, which inhibits triggering receptor expressed on myeloid cells 2 (TREM2) and activates STING, enhanced macrophage phagocytosis and CD8+ T-cell infiltration, illustrating that benefit can involve functional reprogramming rather than cytokine induction alone (19). Responsiveness is not uniform among tumor-associated macrophages (TAMs). The protein phosphatase 2A (PP2A)/striatin 4 (STRN4) complex inhibited MST1/2, stabilized YAP/TAZ, and suppressed STING–type I interferon signaling in tumor-conditioned macrophages; disrupting this axis restored responsiveness in preclinical models (20). Conversely, suppressive macrophage remodeling downstream of chronic tumor-cell STING in chromosomally unstable tumor models is a non-cell-autonomous effect, not evidence that macrophage-intrinsic STING is universally tumor-promoting (10). These findings argue for measuring phagocytosis, interferon responsiveness, and NK/T-cell support rather than relying solely on M1/M2 labels.

Endothelial activation may facilitate leukocyte trafficking but may also contribute to systemic inflammatory toxicity. Fibroblasts and other stromal cells can shape chemokine gradients and physical exclusion. Consequently, bulk increases in interferon-stimulated genes, cytokines, or total immune-cell infiltration do not identify the cellular source responsible for benefit or toxicity. Single-cell, spatial, and lineage-resolved analyses are needed to determine whether a treatment engages antigen presentation, macrophage remodeling, vascular activation, stromal reorganization, or direct tumor-cell death (21).

### 3.2 Tumor-cell pathway competence

Tumor-cell-intrinsic STING can support immunogenicity, but the pathway is frequently altered during tumor evolution. In KRAS-driven lung cancer, LKB1 loss was associated with STING suppression and immune evasion (22). STING signaling is also deregulated in subsets of colorectal carcinoma, constraining responses to cytosolic DNA and DNA-damaging therapy (23).

Some defects are reversible. Treatment with the KDM5 inhibitor CPI-455 increased STING expression in MC38 and CT26 colorectal cancer cells *in vitro*. In an MC38 syngeneic tumor model, the *in vivo*-compatible KDM5 inhibitor CPI-48 suppressed tumor growth in a tumor-cell-intrinsic STING-dependent manner (24). Decitabine restored cGAS-STING expression and DNA responsiveness in cancer cells, including breast cancer models. The study also reported increased endogenous retroviral transcripts and intracellular double-stranded RNA, consistent with additional engagement of RNA-sensing pathways (25). Thus, an increased interferon signature after epigenetic therapy does not by itself establish STING-specific rescue. Paired DNA- and direct STING-agonist stimulation could help distinguish upstream cGAS defects from downstream signaling failure, with viability controls and pathway-specific perturbations needed to interpret ex vivo assays.

These findings suggest that pathway competence should be assessed in both malignant cells and host immune compartments. STING expression alone is insufficient: subcellular localization, TBK1-IRF3 competence, inducible interferon output, negative regulators, and the distribution of responsive cells may all be relevant. None is currently a validated stand-alone predictive biomarker for STING agonist therapy.

## 4 Chromosomal instability: a defined context for chronic signaling

Chromosomal instability (CIN) provides the clearest example of why endogenous chronic signaling must be distinguished from pharmacological agonism. Bakhoum and colleagues showed that CIN can drive metastasis through a tumor-cell-autonomous cytosolic DNA response (9). Li and colleagues subsequently demonstrated that CIN-associated chronic STING signaling can remodel the tumor microenvironment through non-cell-autonomous inflammation and that STING inhibition can suppress CIN-driven metastasis in experimental models (10). The appropriate conclusion is deliberately narrow. These studies support a role for CIN-associated chronic tumor-cell-intrinsic STING signaling in metastatic progression in the models tested. They do not show that all CIN-high tumors use the same program, that every form of STING activation promotes metastasis, or that pharmacological agonists are intrinsically tumor-promoting. CIN should instead be regarded as a candidate stratification variable that may identify tumors requiring closer assessment of the signaling compartment, downstream output, and treatment schedule. STING inhibition in this setting remains a preclinical hypothesis that requires prospective validation, pharmacological selectivity, and safety assessment.

## 5 Tumor genotype and immune state

### 5.1 dMMR/MSI-H and pMMR/MSS colorectal cancer

The response of colorectal cancers with deficient mismatch repair (dMMR) or high microsatellite instability (MSI-H) to PD-1 blockade is multifactorial. MMR deficiency generates a high mutation burden and an expanded neoantigen landscape and is itself a strong, clinically validated predictor of ICB benefit (26–28). Pre-existing immune infiltration and other features of the tumor microenvironment also contribute. Endogenous DNA sensing and type I interferon signaling may complement these processes by supporting immune surveillance, but they should not be presented as the principal explanation for the efficacy of PD-1 blockade.

Proficient mismatch repair (pMMR) or microsatellite stable (MSS) colorectal cancers are similarly heterogeneous. Some are immune inflamed yet checkpoint resistant; others are immune excluded, stroma-rich, or CIN-high; and some have impaired tumor-cell STING signaling (29). Testing STING agonists across these states as a single histological group risks obscuring a responsive subgroup. A weak aggregate clinical signal may reflect inadequate biological stratification, but it may also reflect insufficient drug delivery or pathway-independent immune resistance. These alternatives should be distinguished experimentally rather than inferred from response alone.

### 5.2 A context-based view across solid tumors

Across tumor types, immune state may be a more informative starting point than histology alone. An immune-inflamed, antigen-rich, interferon-competent tumor may be positioned for STING-mediated reinforcement or combination with ICB. An immune-desert tumor with intact antigen presentation but insufficient innate priming may require targeted delivery together with a treatment that promotes antigen release. A tumor with epigenetically silenced or genetically impaired STING signaling may still respond through host immune cells, but tumor-cell expression cannot be assumed to indicate pathway competence. A CIN-high tumor with persistent tumor-cell-intrinsic signaling requires particular caution because additional agonism, host-cell activation, and pathway inhibition are biologically distinct interventions (10).

Concrete examples illustrate different bottlenecks. In breast cancer models, ectonucleotide pyrophosphatase/phosphodiesterase 1 (ENPP1) degraded extracellular cGAMP and limited paracrine activation of host STING; selective loss of its cGAMP-hydrolyzing activity restrained tumor growth and metastasis (30). A macrophage-directed strategy using GB2 also retained activity in a STING-low mouse melanoma model (19). Together with LKB1-associated suppression in KRAS-driven lung cancer (22), these findings suggest that deficient ligand availability, malignant-cell silencing, and suppressive myeloid states should not be grouped as one “STING-low” phenotype. These remain preclinical rationales for stratification, not histology-independent treatment indications.

## 6 Clinical translation of STING agonists

Early clinical studies confirm that intratumoral STING agonists can engage the pathway, but they have not established a broadly effective therapeutic strategy. In a first-in-human study of MIW815 (ADU-S100), 47 patients received intratumoral monotherapy. The agent was generally tolerable and produced evidence of peripheral immune activation, but objective activity was limited to one confirmed partial response (31). In a phase Ib study of MIW815 plus the PD-1 inhibitor spartalizumab in 106 patients, on-target pharmacodynamic activity was observed and the overall response rate was 10.4%, but antitumor activity remained limited (32).

Ulevostinag showed dose-dependent exposure, transient cytokine induction, and manageable toxicity in a 156-patient phase I study. A small randomized phase II cohort in recurrent or metastatic head and neck squamous-cell carcinoma reported complete or partial responses in four of eight participants receiving ulevostinag plus pembrolizumab and one of ten receiving pembrolizumab alone; the sample was too small to establish comparative efficacy (33). In a 24-patient phase I study of E7766, interferon-related pharmacodynamic changes were observed; eight patients achieved stable disease as their best response, and no objective response was reported (34). Together, these findings separate target engagement from clinical benefit and support evaluation in more homogeneous, biomarker-defined populations.

Clinical development should therefore incorporate serial tissue and blood sampling, comparisons of injected and uninjected lesions, and explicit assessment of pathway competence. Studies should distinguish failure of delivery from failure of immune priming, and failure of priming from downstream T-cell exclusion or exhaustion. Recent reviews of cancer-associated cGAS-STING biology, colorectal cancer, and delivery strategies provide complementary perspectives on these translational challenges (14, 21, 29).

## 7 Candidate biomarkers and a framework for patient selection

The early-phase trials discussed here have not established a prospectively validated predictive biomarker for STING agonists (31, 32, 34). dMMR/MSI-H versus pMMR/MSS status, tumor mutational burden (TMB), CIN, baseline interferon activity, STING expression, and immune infiltration should therefore be described as candidate biomarkers. Their value is likely to be combinatorial rather than independent. A practical development framework should connect each candidate measurement to a biological question and specify the compartment being measured. **Table 1** summarizes these candidate variables and their principal interpretive limitations.

**Table 1:** Candidate variables for STING-targeted therapy.

Biological dimension	Candidate assessments	Interpretive use and limitation
Antigenicity and established ICB context	dMMR/MSI-H versus pMMR/MSS status, TMB, and neoantigen burden	Defines antigenic context. dMMR/MSI-H predicts ICB benefit, but none of these measures independently predicts response to a STING agonist.
Genome instability and endogenous signaling	CIN score, micronuclei, cytosolic DNA, tumor-cell cGAS-STING output	May identify chronic tumor-cell-intrinsic signaling; requires spatial and functional confirmation.
Pathway competence	STING localization and expression, stimulus-induced TBK1-IRF3 activation, and type I IFN output	Expression alone is insufficient; functional competence should be assessed in both malignant and host immune compartments.
Immune topology	Dendritic cells, CD8+ T cells, macrophage states, stromal exclusion	Helps distinguish deficient priming from downstream exclusion or suppression.
Kinetics and delivery	Lesion accessibility, drug exposure, injected/uninjected lesion sampling, and tissue/blood pharmacodynamics	Separates delivery and target engagement from durable antitumor immunity.
Prior treatment and adaptive state	Baseline or chronic IFN signatures, prior ICB or radiotherapy, resistance programs	Hypothesis-generating; chronic IFN signatures are not necessarily STING specific.

Candidate development variables; none is a validated stand-alone predictor of response to STING-targeted therapy. CIN, chromosomal instability; dMMR, deficient mismatch repair; ICB, immune checkpoint blockade; IFN, interferon; MSI-H, microsatellite instability-high; MSS, microsatellite stable; pMMR, proficient mismatch repair; TMB, tumor mutational burden.

An actionable panel could combine baseline, compartment-specific cGAS/STING localization with agonist-induced TBK1 and IRF3 phosphorylation, followed by interferon output. Spatial analysis should resolve cDC1–CD8+ T-cell proximity and macrophage functional states; the cDC1 requirement for PolySTING activity provides preclinical justification for interrogating this compartment (17). An exploratory analysis of the I-SPY 2 cohort associated tumor ENPP1 expression with outcomes after pembrolizumab-containing neoadjuvant therapy, but this does not validate ENPP1 for selecting patients for STING agonist treatment (30). On-treatment cytokine changes should be treated as pharmacodynamic evidence, not response predictors: E7766 induced interferon-related changes without objective responses in its phase I study (34). Assay thresholds and sampling times should be prespecified, with predictive value tested against a comparator rather than inferred from within-arm associations.

Prospective trials should predefine composite hypotheses rather than retrospectively search broad marker panels. For example, an agonist trial could require an intact host antigen-presenting cell compartment, documented pharmacodynamic activation, and a defined immune topology while stratifying by CIN and dMMR/MSI-H versus pMMR/MSS status. Patient-derived organoids, immune-organoid co-cultures, and spatially resolved models linked to clinical response may help prioritize combinations, but these models also require validation against intact human immunity. The proposed decision pathway is outlined in **Figure 2**.

**Figure 2:**
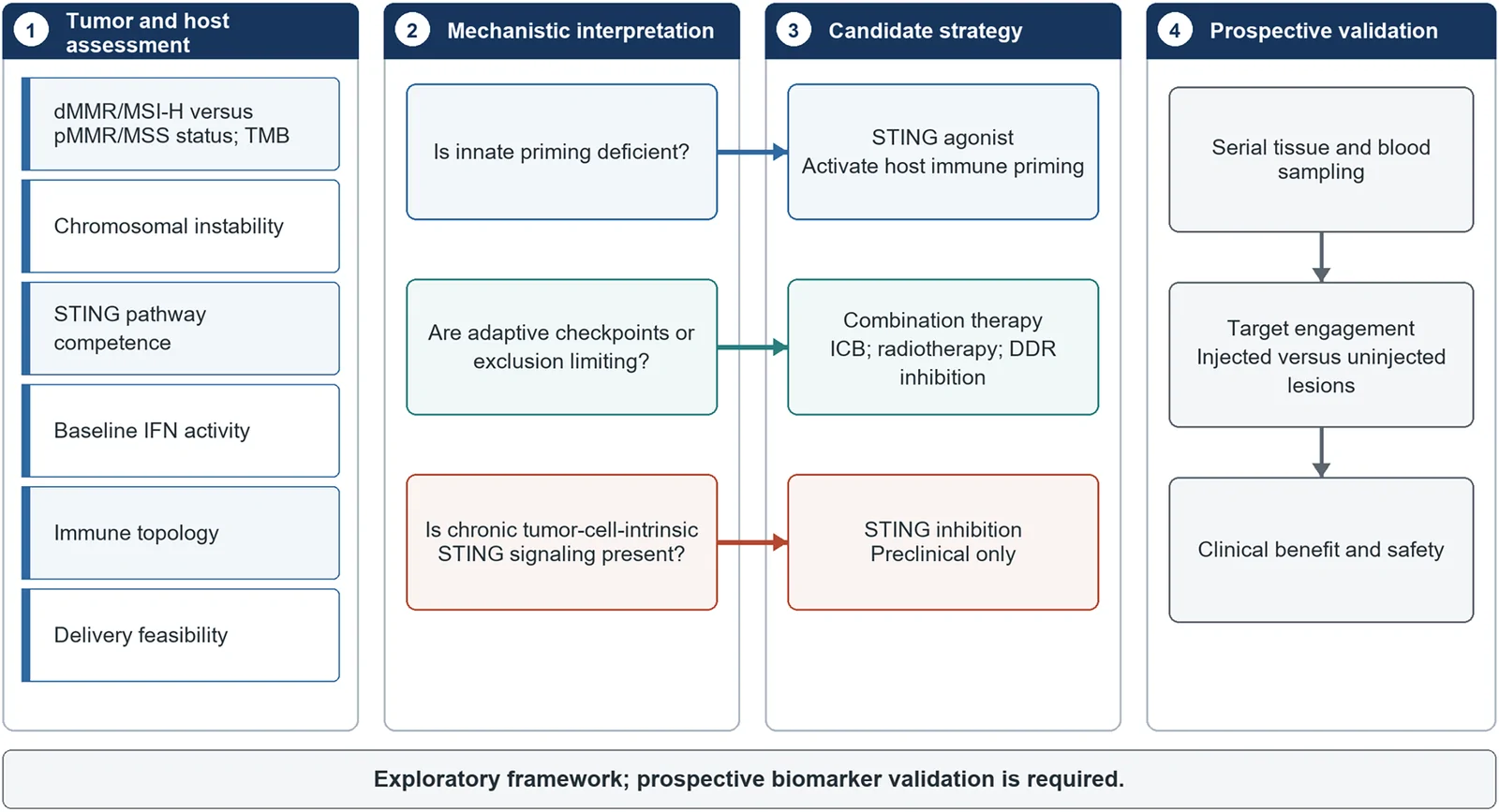
Proposed workflow for biomarker-guided development of STING-targeted therapy. Candidate measurements are interpreted mechanistically before treatment selection. The biomarkers require prospective validation, and STING inhibition remains preclinical. DDR, DNA damage response; dMMR, deficient mismatch repair; ICB, immune checkpoint blockade; IFN, interferon; MSI-H, microsatellite instability-high; MSS, microsatellite stable; pMMR, proficient mismatch repair; TMB, tumor mutational burden.

## 8 Combination strategies and the limited case for STING inhibition

Combination regimens should be selected according to the limiting biological step. ICB may be appropriate when STING activation generates immune priming but adaptive checkpoints constrain effector function. However, the limited activity of MIW815 plus spartalizumab and the small ulevostinag–pembrolizumab cohort preclude assuming that pharmacodynamic activation predicts combination efficacy (32, 33). Radiotherapy, chemotherapy, or DNA damage-response inhibitors may increase antigen release and cytosolic DNA, but they may also intensify chronic signaling or normal-tissue toxicity. Schedule and compartmental exposure are therefore critical, and combinations should not be justified solely by additive inflammatory biomarkers.

Mechanistic support is more specific in small-cell lung cancer models: poly(ADP-ribose) polymerase (PARP) inhibition plus radiotherapy increased CCL5/CXCL10 through cGAS-STING activation, with concomitant downregulation of EIF4E2 enhancing CXCL10 mRNA stability; adding PD-L1 blockade improved T-cell infiltration and tumor control (35). This regimen activates endogenous DNA sensing and does not establish that adding an exogenous STING agonist will provide further benefit. Macrophage reprogramming with GB2 also enhanced PD-1 blockade in mice, supporting combinations that relieve a defined myeloid barrier (19).

STING inhibition should be considered separately. The strongest rationale is suppression of CIN-associated chronic tumor-cell-intrinsic signaling in experimental models (10). This remains a promising preclinical hypothesis rather than an established therapeutic strategy for CIN-high tumors. Clinical translation will require selective inhibitors, biomarkers that distinguish maladaptive tumor-cell signaling from beneficial host immunity, and evidence that inhibition does not compromise immune surveillance. Because macrophage STING supported NK-mediated control of colorectal liver metastasis (18), nonselective inhibition could remove beneficial host signaling. Tumor-restricted delivery and compartment-resolved pharmacodynamics are therefore priorities for testing an inhibitory strategy, not established solutions.

## 9 Conclusions

STING is not a binary therapeutic switch. Acute pharmacological activation of immune cells and chronic endogenous tumor-cell-intrinsic signaling are biologically distinct, and evidence from the latter should not be used to infer that STING agonists are broadly tumor-promoting. The clinical task is to define the responsive compartment, signaling duration, downstream output, tumor genotype, immune topology, and delivery profile. Current biomarkers remain candidates rather than validated predictors, and STING inhibition in CIN-high tumors remains preclinical. Compartment-resolved, biomarker-driven trials are needed to determine when STING should be activated, combined with another therapy, or restrained.
